# Bayesian Algorithm Implementation in a Real Time Exposure Assessment Model on Benzene with Calculation of Associated Cancer Risks

**DOI:** 10.3390/s90200731

**Published:** 2009-02-02

**Authors:** Dimosthenis A. Sarigiannis, Spyros P. Karakitsios, Alberto Gotti, Costas L. Papaloukas, Pavlos A. Kassomenos, Georgios A. Pilidis

**Affiliations:** 1 European Commission (EC), Joint Research Center (JRC), Institute for Health and Consumer Protection (IHCP), Physical and Chemical Exposure Unit (PCE), Ispra (Va), I-21020, Italy; E-Mails: spyridon.karakitsios@jrc.it (S.K.); alberto.gotti@jrc.it (A.G.); 2 University of Ioannina, Department of Biological Applications and Technologies, Laboratory of Bioinformatics, GR-45110, Ioannina; E-Mail: papalouk@cc.uoi.gr (C.P.); 3 University of Ioannina, Department of Physics, Laboratory of Meteorology, GR-45110, Ioannina; E-Mail: pkassom@uoi.gr (P.K.); 4 University of Ioannina, Department of Biological Appl. and Technologies, Laboratory of Environmental Chemistry, GR-45110, Ioannina; E-Mail: gpilidis@uoi.gr (G.P.)

**Keywords:** Bayesian algorithm, ANN, PBPK, benzene exposure

## Abstract

The objective of the current study was the development of a reliable modeling platform to calculate in real time the personal exposure and the associated health risk for filling station employees evaluating current environmental parameters (traffic, meteorological and amount of fuel traded) determined by the appropriate sensor network. A set of Artificial Neural Networks (ANNs) was developed to predict benzene exposure pattern for the filling station employees. Furthermore, a Physiology Based Pharmaco-Kinetic (PBPK) risk assessment model was developed in order to calculate the lifetime probability distribution of leukemia to the employees, fed by data obtained by the ANN model. Bayesian algorithm was involved in crucial points of both model sub compartments. The application was evaluated in two filling stations (one urban and one rural). Among several algorithms available for the development of the ANN exposure model, Bayesian regularization provided the best results and seemed to be a promising technique for prediction of the exposure pattern of that occupational population group. On assessing the estimated leukemia risk under the scope of providing a distribution curve based on the exposure levels and the different susceptibility of the population, the Bayesian algorithm was a prerequisite of the Monte Carlo approach, which is integrated in the PBPK-based risk model. In conclusion, the modeling system described herein is capable of exploiting the information collected by the environmental sensors in order to estimate in real time the personal exposure and the resulting health risk for employees of gasoline filling stations.

## 1. Introduction

Employees in fuel stations are exposed to high levels of gasoline vapours (including benzene) during refuelling, as well as high concentrations of emissions from tail pipe exhausts. Therefore, service station employees are a vulnerable sector of the population because of their exposure during their 8 h shifts [1], especially considering the well known health effects focused mainly on increased risk of leukaemia after chronic exposure and neurotoxic effects after short-term exposure to high values of benzene vapour and other volatile organic chemicals present in this environmental setting [2].

Until now, several studies were performed determining the personal exposure of filling station employees [3-5]. Most of the studies related to the employee exposure were limited to assessing exposure to benzene only over a time shift (8 h duration). Assuming that the dominant source of exposure is fuel evaporation, studies of exposure determinants have focused on empirical relations among the amount of fuel traded and ambient temperature and the results were more qualitative than quantitative [6]. In recent years, after the adaptation of Stage II fuel evaporation recovery system in fuel pumps, exposure to benzene was significantly reduced (up to one order of magnitude). As a result, the effect of exposure parameters other than refuelling such as urban pollution levels and contribution of adjacent streets was more decisive, making the quantification of the exposure determinants more complicated.

The concept proposed in the present study is based on the concerns mentioned above. The overall aim is the development of a monitoring-modelling system able to evaluate in real time the continuously changing values of the environmental parameters (detected by sensors) that affect public and occupational health risk. In order to achieve this, besides setting up an adequate sensoring network for the relevant environmental parameters, the development of the proper algorithms for evaluating and processing the acquired data is necessary. The main sub compartments of the modelling scheme are the exposure model which is an ANN model, which in turn feeds the risk assessment model, which is based on a biological description of the biokinetics and the mechanisms of action of the main environmental toxicants of concern in this study.

ANNs have been largely used to predict atmospheric pollutant concentrations, especially the reactive ones like NO_X_ and O_3_ [7], as well as for several other kinds of environmental applications [8]. The main reason for this is their remarkable ability to handle non linear systems. The non reactive pollutants are also non linear systems (in a much smaller degree than photoreacting pollutants) since they are highly depend on atmospheric dispersion conditions and vehicles emissions. Recently, non reactive pollutants forecasting via ANNs [9, 10], has gained an increased interest because ANNs can handle the large amounts of data now routinely acquired through modern monitoring techniques. Yet, according to our knowledge, ANNs have not been used so far for the prediction of human exposure. In the present study, the developed set of ANNs aims to produce a model predicting exposure and quantifying the contribution of the exposure pattern determinants.

After the determination of exposure and the related parameters, the potential health implications were assessed through a PBPK/PD model developed ad hoc. The concept of the model lies on the determination of concentrations of benzene metabolites (catechol, phenols, muconic acid, phenylmercapturic acid and hydroquinone) on target issues (bone marrow) and, based on the dose response curve, the calculation of the estimated risk, considering also the diversity of the population through a Markov Chain Monte Carlo approach. PBPK models [11] provide biology-based knowledge of the mechanisms of chemical toxicity; this is a more rational and sophisticated way for estimating potential health implications than empirical relations extrapolated from epidemiological studies. As such, they can be very valuable tools for improving the physiological and biological basis of regulatory health risk assessment.

## 2. Methodology

### 2.1. General

The aim of the study is the development of a platform that:

- Gathers information from crucial environmental parameters (through the proper environmental sensors), which constitute determinants of the exposure to benzene of filling employees
- Implements the proper algorithms evaluating the information obtained by the environmental sensors, in order to provide real time estimation (a) of exposure to benzene and consequently (b) of the associated health risk.

**Figure 1.**
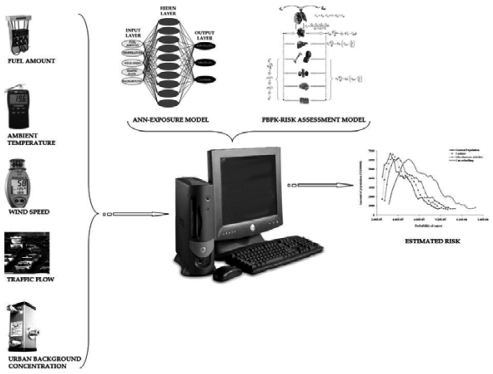
Visual concept of the proposed exposure and risk assessment platform.

As a result, the main interest of the present study is in the development of the modelling schemes that utilize the information gathered by the sensors. For this purpose, two individual modelling concepts where adopted: one for the calculation of exposure to benzene (an ANN based model) and one for risk estimation due to exposure to benzene (a PBPK/PD model). A visual presentation of the whole platform is presented in Figure 1.

Beside the theoretical complexity of the study, both sub-models were developed and validated based on results of experiments, including environmental field measurements (ANN model) as much as laboratory studies (PBPK model). Thus, the field measurements campaign constitutes a significant part of the whole study and the adopted techniques.

### 2.2. Monitoring sensor network design

**Figure 2.**
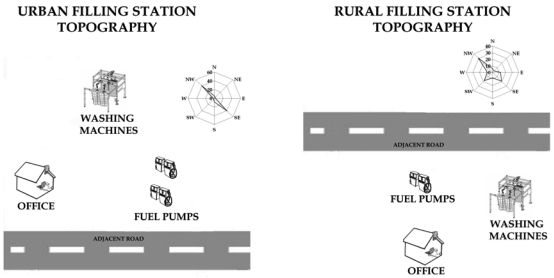
Topography of the filling stations in combination to the prevailing wind.

The sensor network developed in this study was deployed experimentally in Ioannina, a medium sized (nearly 100,000 inhabitants) city in Northwestern Greece, with relatively high air pollution values [12]. This network comprised sensors for monitoring urban background concentrations of ambient air (multi-pollutant sensors), traffic flow meters, wind speed sensors, digital thermometers, and fuel gauges. Given that near the wider area no significant industrial activity is observed, the dominant source of ambient air pollution is traffic, contributed by central heating during winter. The measurements campaign was performed during two sampling periods (August and December, 2006) to investigate the seasonal variation of benzene exposure values. The study was performed at two filling stations, an urban and a rural one, at a distance of 15 km of the city limits. Both of them were equipped with Stage II fuel evaporation recovery systems. The location of the stations and the adjacent roads, in relation with the prevailing wind direction are described in Figure 2. Personal exposure to benzene by passive sampling was measured with 15 volunteers each season for two weeks during the working hours (8:00-16:00). One sampler per day was used for each volunteer. The volunteers group included 10 employees in the urban filling station and five in the rural one. People working in filling stations were doing three major activities: car refuelling, miscellaneous activities (like car washing or oil checking) and managing and operating the cash machine inside the filling station office. Most of the employees were doing a combination of these activities, especially in the rural filling station. In order to examine the daily exposure variation and the significance of the activities in the configuration of the total amount of exposure for the filling station employees, active sampling was also performed. In this case, sampling pumps were attached on the employees, and the samplers were placed at chest height, similarly to the passive samplers.

This was attainable due to the relatively small size and weight of the sampling pumps. The employees carrying the pumps executed strictly only one kind of activity at a time, classified as refueling, cashier work and miscellaneous. The pump was set to work for 30 minutes at a flow rate of 100 mL/min. Each sample was attached for half an hour, and one sample was used per hour; thus by the end of the 8-hour shift, eight samples were taken per employee. In each filling station, one employee for each activity class carried the active sampler. Active sampling was performed daily. In total, 336 samples were taken from each gasoline station.

Apart from active sampling, the following parameters were measured, and all the values were recorded continuously and registered electronically every 30 minutes:

- The amount of the gasoline that was traded.
- Wind speed (m/sec) and direction (degrees).
- Ambient air temperature.
- Traffic flow from two independent observers. Vehicles were registered in seven main categories (catalytic passenger vehicles, non catalytic passenger vehicles, diesel passenger vehicles, light duty passenger vehicles, heavy duty passenger vehicles, buses and motorcycles) and the traffic volume per category was recorded every half an hour. In the urban site the measurements were continuous from 8:00 to 16:00. Vehicle speed was calculated by the quotient of the distance (a part of the road) and the time needed to cover that distance.
- Urban background concentration. The most common and reliable modelling practice to define background concentration is to use data obtained from measurements at urban locations that are not directly affected by local sources [13]. Based on this, two active samplers were placed in two different urban locations (not affected by traffic or other known benzene sources) and their values were averaged in order to exclude the urban background concentration. In the rural area also two passive samplers were placed to investigate any possible background concentration or the existence of any other benzene source beside the road and the gas station emissions that may affect the measurements results. In the operational mode of a multi-sensor fusion based monitoring system as the one outlined in this study, these parameters would be measured using automated sensors and their output data streams integrated following exactly the same algorithm as the one given in this paper.

The equipment used in the monitoring sensor network and the related analyses is listed below:

- Varian 3900 GC gas chromatography system with a flame ionization detector (FID). The capillary column through which the chromatographic separation of the various pollutants was effected, is the 30 m long, 0.53 mm inner diameter and 0.5 μm film thickness, SBM™ -5 capillary column, by Supelco, Italy.
- MARKES Thermal Desorption Cold Trap Injector thermal desorption system
- Three low volume SKC model 222 pumps for gas sampling
- DryCal CD-Lite (Bios International, USA) flow meter with a measurement range of 0.010 to 12 L/min,
- Sampling tubes (suitable both for active and passive sampling): MARKES CARBOGRAPH 5TD tubes standard absorbing cartridges filled with 400 mg of sorbent.
- Qualimetrics model 2020 and 2032 cup anemometer and wind vane, respectively.

The quality assurance and quality control (QA/QC) procedure included laboratory and field blanks, parallel samples and duplicate measurements of samples. Before each sampling with a canister, blank sample (zero air) was analyzed to ensure that the concentration of all compounds inside was below 0.02 ppbv. Parallel samples and duplicate measurements of samples were analyzed to test the precision of the sampling and analytical techniques, respectively. The mean relative standard deviations (RSD) for all the compounds were less than 8%. A new calibration curve was determined each time. The detection limit of each compound was calculated from the data of seven replicate measurements of low concentration samples and observed from their standard deviation. The correlation coefficient for benzene was 0.9983. Response linearity tests showed that the response signal was proportional to injection volume for benzene concentrations (0.5–220 μg/m^3^).

### 2.3. ANN (Artificial Neural Network) modeling development

In order to predict the exposure pattern of the filling station employees, a set of ANNs was developed. The input parameters used were (i) the amount of gasoline sold, (ii) the traffic flow of the road in front of the filling station, (iii) the background concentration (only in the urban filling station because in the rural it was almost zero), (iv) the wind speed, and (v) the ambient temperature, the ANN output was the exposure pattern of the three main subcategories of employees mentioned above. Consequently, three ANNs were employed, one for each group of workers. The ANNs can provide significant information for environmental policy purposes, after quantifying the parameters that constitute the exposure pattern, in terms of the Relative Importance (% contribution values). In their general form, ANNs refer to a parallel model architecture able to perform numerical calculations based on distributed processing. ANNs are composed from artificial neurons that operate according to a specified transfer function. The neurons interact with each other through the use of neuron connections called weight factors or simply weights. Positive or negative weights correspond to connections that propagate or suspend, respectively, signals from other neurons. ANNs are trained to perform a particular function by adjusting properly the values of these weights.

**Figure 3.**
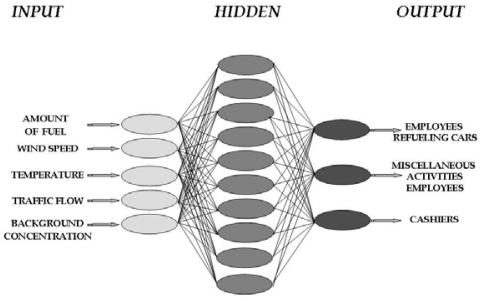
Structure of the proposed ANN.

In this approach a feedforward network architecture was adopted. This is a multilayer architecture, with an input layer, where data are introduced to the ANN, one or more hidden layers, where processing is realised, and an output layer, which generates the final results (Figure 3). Another important characteristic of feedforward ANNs is that neuron activation (or signalling) propagates towards one direction only and specifically from the input layer to the hidden layers and finally to the output layer.

In this study, three different sets of ANNs were developed: one for the urban station, one for the rural and one for both. The number of the neurons corresponds to each input parameter. The input parameters were the amount of gasoline traded, wind speed, ambient temperature and traffic flow of the road in front of the filling station and benzene urban background concentration. The difference between the first and the other two sets of ANNs is that for the urban station the input layer consisted of five neurons while for the rural one consisted of four, due to the lack of background concentration. In the third set, data gained from both stations were used for training and testing the ANN. In this case, the lack of the background concentration as an input parameter was not very determining for the urban stations' predictions, because it was indirectly included in the parameters of traffic flow and wind speed. We should note that in all cases the ANN architectures were the same. Specifically, the input layer had four or five nodes, the hidden layer 10 and the output layer three (Figure 3).

In particular, the second (hidden) layer consists of 10 neurons that implement the hyperbolic tangent sigmoid transfer function. This function generates outputs between -1 and 1 as the neuron's net input goes from negative to positive infinity and is more appropriate for ANN applications on function approximation. It should also be noted that the input parameter values are pre-processed in order to be normalized according to Eq. 1, the inputs and targets will fall in the interval [-1, 1].

(1) $$x_{n}=2\frac{x-\min(x)}{\max(x)-\min(x)}-1$$

As for the number of the hidden neurons, various architectures were tested (different numbers of neurons and hidden layers) but the choice of one hidden layer with 10 neurons provided consistently the best results. Finally, the third (output) layer consists of three linear neurons that correspond to the three predicted exposure concentrations for each one for each exposed group of workers.

Network training was performed using the Bayesian regularisation algorithm [14-15]. This is a supervised learning method that determines the optimal network parameters in terms of model generalisation. These parameters, namely the weights and biases of the network, are assumed to be random variables with specified distributions, with prior and posterior probabilities given by equations (2) and (3):

(2) $$P(\text{w}|\alpha_{1},A,R)=\frac{\exp[-\alpha_{1}E_{W}(\text{w}|A)]}{Z_{W}(\alpha_{1})}$$

where **w** are the network parameters, *a*_1_ is a regularising constant, *A* is the network architecture, *R* are the alternative regularisers with different energy function *E_W_* each and *Z_W_* = ∫ *d^k^***w**exp[−*a*_1_*E_W_*],

(3) $$P(\text{w}|\alpha_{1},\alpha_{2},A,R)=\frac{\exp[-(\alpha_{1}E_{W}+\alpha_{2}E_{D})]}{Z_{M}(\alpha_{1},\alpha_{2})}$$

where *α_2_* is a regularising parameter and *Z_M_*(*α*_1_,*α*_2_) = ∫ *d_k_***w**exp[−(*α*_1_*E_W_* + *α*_2_*E_D_*)]. The regularization parameters are related to the unknown variances associated with these distributions. Consequently, based on statistical techniques these parameters can be estimated.

Within this framework the following error function is minimised:

(4) $$E=a_{1}\sum_{i=1}^{N}{\left(t_{i}-o_{i}\right)}^{2}+a_{2}\sum_{i=1}^{M}{\text{w}}_{i}^{2}$$

where *t_1_* are the desired network outputs, *o_i_* are the network outputs during training, **w***_i_* are the network parameters (weights and biases), *M* is the number of those parameters and *N* is the number of the training patterns. The hyperparameters *a_i_* are estimated at each iteration according to the following formulas:

(5) $$a_{1}=\frac{N-\gamma }{2\sum_{i=1}^{N}{\left(t_{i}-o_{i}\right)}^{2}}$$

(6) $$a_{2}=\frac{\gamma }{2\sum_{i=1}^{M}{{\text{w}}_{i}}^{2}}$$

where *γ* is the number of effective parameters and is given by:

(7) $$\gamma =N-2a_{2}\text{tr}{\left(\text{H}\right)}^{-1}$$

with **H** being the Hessian matrix of the error function and can be approximated as follows using the Jacobian matrix, **J**, that contains the first derivatives of the network errors with respect to the weights and biases:

(8) $$\text{H}={\text{J}}^{T}\text{J}$$

The network parameters **w**_*i*_ are updated according to the Levenberg-Marquardt optimisation method:

(9) $${\text{w}}_{i+1}={\text{w}}_{i}-{\left[{\text{J}}^{T}\text{J}+\mu \text{I}\right]}^{-1}\text{g}$$

where **I** is the unit matrix, μ is a scalar parameter [16] and **g** is the gradient which can be computed as:

(10) $$\text{g}={\text{J}}^{T}\text{e}$$

with **e** being the vector of network errors.

The network parameters are initialised using the Nguyen-Widrow method [17] and the two hyperparameters, *a*_1_ and *a*_2_, are initially set to one and zero, respectively. Apparently, the error function *E* is adapted at each iteration, since the hyperparameters *a_i_* are re-estimated. According to the Bayesian regularization algorithm the training procedure is considered to be completed when the effective number of parameters, *γ*, has converged, indicating that the ANN has generalised and not overfitted the training data.

Besides Bayesian regularization, the performance of the proposed methodology using various training algorithms on different network architectures was also evaluated. Specifically, algorithms from simple variants of the well known backpropagation algorithm like Resilient Backpropagation [18] and Scaled Conjugate Gradient [19] to more sophisticated ones like BFGS [20] and Levenberg-Marquardt [16] were employed.

For training and testing the proposed ANN model we followed the procedure of Leave-One-Out Cross-Validation (LOOCV). According to this schema, iteratively one sample is kept for testing while the rest are used for training the model, until all samples are finally tested [21]. There are many ways to implement cross-validation, however LOOCV is a very commonly used validation schema and is considered to better resemble real practice where the proposed model is trained with all the samples at hand and then is applied to the specific application.

### 2.4. PBPK-based risk assessment model

Physiologically based pharmacokinetic (PBPK) models propose a realistic even if simplified description of the mechanisms of absorption, distribution, metabolism and elimination of chemicals in the body. In these models, the body is subdivided into various compartments representing specific organs or homogeneous groups of tissues linked and irrigated by blood vessels. Compartments are characterised by a set of parameters of physiological relevance (e.g., volume or blood perfusion rate) which play a crucial role in explaining the behaviour of chemical substances in the body, and represent invariants across substances. Physiologically based pharmacokinetic (PBPK) models offer great flexibility. In particular, they provide a parametric framework suitable for dealing with extrapolations between species, routes or dose levels [11, 22-23].

The PBPK model for a generic mixture of chemicals is represented as a combination of “single chemical” models interconnected at level of hepatic metabolism where the effect of the interaction is evaluated according to the potential mechanism of action (competitive, noncompetitive, and uncompetitive metabolic inhibitions). The latter, in the case of a BTEX mixture, is assumed to be of “competitive inhibition” since the four VOC's considered are known substrates for the same cytochrome P_450_ isozyme (CYP2E1). This was confirmed by the analysis of the kinetic data from all binary exposure studies relevant to the BTEX mixture [24]. The models for toluene, ethylbenzene and all the family of xylenes are all four-compartment models encompassing richly perfused tissues (RPT), poorly perfused tissues (PPT), adipose tissues (FAT), and liver (metabolising tissue), interconnected by systemic circulation and a gas exchange lung.

The model for benzene is a six-compartment model. The six tissue groups include: liver (main metabolic tissue); adipose tissue (FAT); richly perfused tissues (RTP); poorly perfused tissues (PPT); bone marrow (the main target organ for benzene toxicity) and the kidney; each one interconnected to the others by systemic circulation and a gas-exchange lung. The bone marrow was included because it is recognised as the main site manifesting benzene toxicity (i.e., leukaemia) and because it is, together with the kidney, a potential site benzene metabolism. The liver was further subdivided into three equal volume sub-compartments according to the zonal distribution of enzymes that mediate benzene metabolism [25]. Metabolism mediated by CYP2E1 is assumed to occur by and large in “zone 3” of the liver; sulfation takes place primarily in “zone 1”; non-enzymatic metabolism occurs in all the three compartments as well as in all tissues.

The high potential toxicity of benzene metabolites associated to the leukemia risk in humans, suggested taking into account more in detail the metabolic chain from benzene to its key metabolites through a more refined PBPK model for that chemical. The whole metabolic chain of benzene was modelled starting from previously developed PBPK models for benzene metabolism in mice [25] and its extrapolation to humans [26], which in turn where based on experimental data of individuals exposed to benzene.

**Figure 4.**
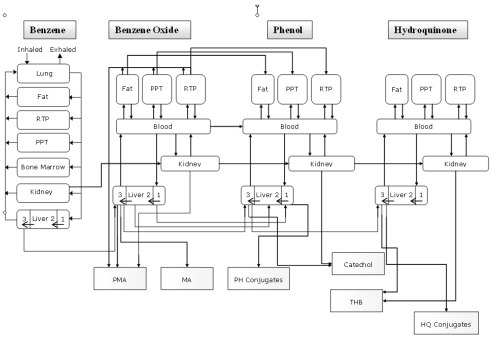
Conseptual representation of the PBPK model.

The model (Figure 4) evaluates tissue levels of benzene, benzene oxide (BO), phenol (PH), and hydroquinone (HQ), as well as the total amounts of muconic acid (MA), phenylmercapturic acid (PMA), phenol conjugates, hydroquinone conjugates, and total catechol produced.

For benzene oxide, phenol, and hydroquinone, the body is divided into five compartments: kidney; liver; fat; rapidly perfused tissues (RTP), and slowly perfused tissues (PPT). As for the benzene model the liver is subdivided into three compartments of equal volume according to the specific enzymatic distribution. The further metabolism of BO, PH and HQ is supposed to occur in the liver (main metabolism organ) and to a lesser extent in the kidney.

More in detail the followings metabolic transformations (mediated by CYP2E1) are supposed to occur in zone 3 of the liver as well as in the kidney:

benzene → benzene oxide
phenol → hydroquinone
phenol → catechol
hydroquinone → trihydroxy benzene

Since the kidney contains approximately 10% of the concentration of CYP2E1 found in the liver [27], it is assumed that relative to the metabolism in the liver, 10% of the metabolism mediated by CYP2E1 is in the kidney.

Having in mind that cancer risk associated to benzene is mainly related to its metabolites and their internal dose, a biology-based approach that takes into account the internal dose of metabolites produced is an improvement compared to traditional risk assessment that provides a more robust biological basis. Furthermore, starting from prior distributions of the most important physiological and biochemical parameters influencing the determination of the internal dose of benzene metabolites we can derive a distribution function of health risk to the exposed population rather than a unique value representing an “individual” risk using Markov Chain Monte Carlo techniques [28]. In particular, starting from literature-obtained prior distributions of key biochemical and physiological parameters known to affect the level of individual susceptibility to xenobiotic health stressors such as age, gender, weight, fat percentage and distribution in the human body we can use Bayesian statistics to calculate posterior distributions of the same parameters in an iterative manner. Following a Markov chain of Monte Carlo simulations the posterior distributions converge to the actual parameter distributions used for estimating the internal and biologically effective dose of the benzene metabolites at the target organs (e.g. the bone marrow). Based on these distributions the related cancer risk is estimated by applying a dose-response (pathology) model for benzene-induced cancer [28]. In this way, the estimated cancer risk is representative not only of an average individual as in the case of most health risk estimates used to date; instead, the resulting risk outcome is expressed in terms of a probability density function of cancer risk for the given population at different exposure levels. In this way, we were able to estimate population risk providing thus a better metric of public health pressure associated to environmental exposure to benzene and its metabolites under realistic conditions taking into account real-time environmental monitoring data. Comparative assessment of health risk using cumulative exposure based on real-time estimations instead of life-average values of benzene exposure has showed that considering time-varying concentrations contributes to a higher precision of the overall health burden compared to using time-averaged concentrations as input. This is certainly true for acute toxicity, but it holds for chronic toxicity as well, especially when personal exposure is characterized by short-term but repetitive high values of benzene. The whole PBPK/PD and pathology (cancer risk) model was developed on acslXtreme simulation software by Aegis Technologies.

## 3. Results and Discussion

### 3.1. Traffic data

Traffic flow and fleet composition (Table 1) on the roads adjacent to the filling stations was measured and traffic speed was calculated. The fleet pattern from the urban station road is dominated by passenger cars equipped with a catalytic converter (70% of the total fleet). The percentage of passenger cars not equipped with a catalytic converter is also significant (8%), followed by motorcycles (7%). Both of them present strong benzene emission potency. The fraction of light-duty trucks is just as high as motorcycles (7%); however, these vehicles are not strong benzene emitters because they run on diesel. In the rural road the fraction of heavy diesel vehicles (trucks and buses) is significantly increased (14%).

Traffic flow in the urban adjacent road presents an average flow of 1,500 veh/h, while for the rural road is about 600 veh/h. In the first case, traffic flow presents maximum at 8:00-9:00 and at 2:00-3:00 related to the transportation of citizens to working places. In the rural road, traffic flow seems to be more uniform, presenting a slight increase at 10:00-11:00. Daily variation of traffic flow is presented in Figure 3, where the average weekly variation is given.

No significant seasonal variability was observed in the urban street, while in the rural road, traffic flow was increased almost to 20% in the summer. Traffic speed in both cases was calculated to be equal to the road limit (50 and 70 km/h respectively), having a deviation of ± 10 km/h.

Traffic data are necessary to estimate the magnitude of benzene's road emission rate and consequently to have an overview of the importance of these emissions to the employees exposure pattern. Based on the daily traffic variation data, benzene emission rate for urban and rural road were calculated, varying from 0.26 - 1.4 gr·km^-1^·sec^-1^ and 0.11 – 0.50 gr·km^-1^·sec^-1^ respectively by using COPERT III methodology [29].

### 3.2. Meteorological data

Wind speed and direction measurements confirmed the historical data on the wind field over the area. The prevailing wind directions, during the sampling periods, were from southwest and northwest directions and are presented in the location maps (Figure 2)

In the urban area, mean wind speed ranged from 0 to 7 m/sec, for the summer period, while the average wind speed was 2 m/sec. In summer, wind speed tended to increase after the evening hours. In the winter, wind speed ranged from 0 to 5 m/sec, having an average value of 1.1 m/sec. In the rural location, wind speed had the same profile, but the mean values where in general increased by a factor 20-30%. From previous studies conducted in the area by the authors it is known that the wind profile varies only mildly during the year. During the spring and autumn wind speed falls on average within the range determined for summer and winter [10]. This provides additional reassurance regarding the efficacy of the ANN in predicting properly the personal exposure values for seasons other than winter and summer.

Ambient temperature in the urban area ranged from 14 to 28 ^°^C, having an average value of 23 ^°^C for summer, while for the winter the corresponding values are -2 to 13 ^°^C and 7 ^°^C respectively. For the rural filling station the values (min-max-average) for summer and winter were 11-26-22 ^°^C and -4-12-6 ^°^C respectively. Temperature variation through the day followed typical patterns for the region, increasing from the morning to the evening.

### 3.3. Amount of gasoline traded

The amount of the gasoline traded during the working hours was recorded automatically by the cash machines which are wired to the fuel pumps in both filling stations.

**Figure 5.**
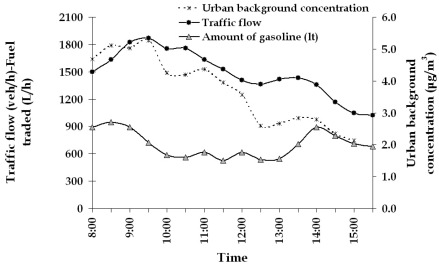
Averaged weekly daily variation of traffic flow (in veh/h), fuel traded (in lt/h) and urban background concentration (in μg/m^3^) of the urban filling station.

In the urban filling station, the amount of gasoline ranged from 550 to 950 L/h, having an average value of 700 L/h, while for the rural station ranged from 150 to 300 L/h, having an average value of 200 L/h. Daily and seasonal variation of the gasoline amount was proportional to the adjacent road traffic flow variation. The averaged weekly daily variation of urban filling station is also presented in Figure 5.

### 3.4. Personal exposure results

**Figure 6.**
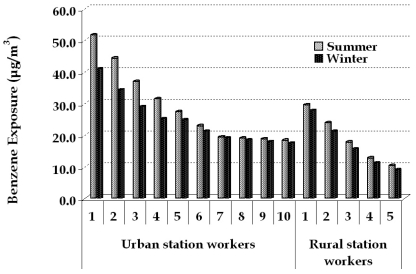
Average weekly benzene exposure (in μg/m^3^) of the filling station employees for summer and winter respectively.

Exposure pattern for the filling station employees is complex, presenting significant variation of the recorded values. In Figure 6 the weekly averaged results of passive sampling are presented. The values ranged from 18.3 to 51 μg/m^3^ in summer and from 17.5 to 41 μg/m^3^ in the winter for the urban filling station. In the rural filling station, the values range from 10.3 to 29.5 μg/m^3^ and 9.1 to 26.8 μg/m^3^ for summer and winter respectively. From a recent exposure survey conducted in the same urban area [30], exposure to benzene for the general population was determined equal to 9 μg/m^3^, thus exposure to benzene for the filling station employees is considered elevated, although the presence of the preventing measures by Stage II fuel evaporation recovery systems adopted.

The higher exposure levels in the urban filling station may be explained by the total amount of gasoline daily distributed (6,000-12,000 liters of gasoline in comparison to 2,000-3,000 liters) and to the absence of any other significant source of benzene in the rural filling station. On the contrary, the emissions from the central road in front and the contribution of the urban background concentration in the urban filling station seem to be significant additive parameters.

A seasonal variation was observed, especially in the urban filling station. The higher temperatures in the summer (average temperature 23 ^°^C, while the respective winter value is 7 ^°^C) explain the increased benzene concentrations in personal exposure values in the vicinity of the filling station due to stronger fuel evaporation. In the urban filling station this observation is more intense, due to the higher amount of gasoline distributed (and consequently evaporated). Furthermore, the corresponding increase in background concentration and direct traffic emissions from the adjacent road, are concurrent to the recorded variation among summer and winter measurements. Results of active sampling measurements were necessary to clarify the importance among the activities occurring in a filling station.

**Figure 7.**
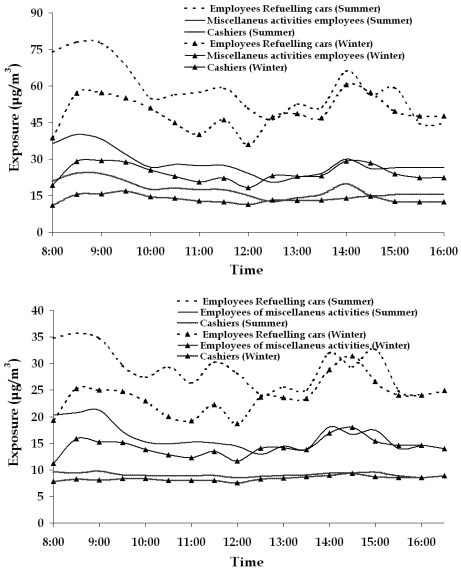
Averaged weekly daily variation of the exposure (in μg/m^3^) for the three categories of employees in summer and winter of urban (a) and rural (b) filling stations respectively.

In Figures 7a-b, the hourly averaged weekly active sampling measurements results for each category of employees are presented. From the results it is evident that employees who actively refuel cars are exposed to the higher benzene levels. This was an expected result because the dominant source of benzene is car refueling.

Daily variation of exposure values of employees working outdoor, seemed to be highly correlated to the amount of gasoline distributed, an observation confirming the results from older studies [6]. The gasoline trading rate is higher in the morning (8-9:00) and evening hours (14-15:00), and it is related to the transportation to/from work. This variational pattern is not observed in the rural filling station, where an increase of the distributed gasoline is noticed only in the morning.

Ambient air temperature also affects significantly the exposure levels. When temperatures are near 0 ^°^C (a fact occurring at the early morning hours in the winter time), the exposure levels are lower than usual for the same amount of fuel traded (Figure 7). The presence of wind seems to reduce exposure levels, especially to employees performing miscellaneous activities. No strong correlation among wind speed and exposure levels of employees refueling cars and cashiers was observed.

Finally, in the urban station, background concentration seems well correlated to the exposure levels of all the categories of employees. The reason for that correlation is the same as for the traffic flow: when the traffic flow is increased, this affects the whole urban area and consequently the urban air pollution is elevated. The quantification of the importance of the above parameters was further investigated through ANN modeling.

Compared to employees refueling cars (Figures 7a-b), employees of miscellaneous activities are exposed to significantly lower benzene concentrations, because they rarely approach the pumps, staying mainly in the area of car washing machines. This means that they work in an average distance of 15-25 meters from the pumps, where the concentrations of benzene are significantly reduced.

The cashiers are working in an indoor environment, which is also affected by the same parameters that configure concentrations in the wider vicinity. Thus, the recorded values are lower than the values of the employees doing other activities. The only difference at the rural filling station (in comparison to the urban one) is the absence of any significant variation among summer and winter exposure measurements of the cashiers. This may have occurred due to an additional indoor source of benzene affecting the concentrations, possibly smoking, which is permitted inside the filling station office.

### 3.5. Artificial Neural Network modelling results

**Figure 8.**
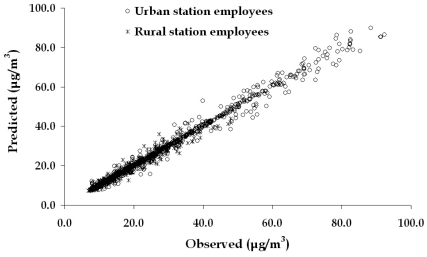
Dispersion among the observed and the predicted values (in μg/m^3^) by the ANN for the exposure of the employees of urban and rural filling station respectively.

The results among the observed and the predicted values are shown in Figure 8. The evaluation parameters RRMSE (Relative Root Mean Square Error) and R^2^ (Correlation) are presented in Table 2.

From the above, it is observed that the performance of the ANN applied to the urban filling station data is slightly better than the ANN applied to the rural one for the first two categories of employees. On the contrary, the performance referring to cashiers is worst in the urban station. This occurs because the indoor concentration of benzene presents a time lag following the outdoor concentrations that determine the exposure of the other two categories of working people and is strongly affected by the general benzene concentrations that affect the wider area. This is also the reason, that among the three categories of employees, the best performance of the model is for the miscellaneous activities employees. The exposure pattern of these employees is directly affected by the referred input parameters, located in the area of the station that does not appear to have acute changes in the observed benzene concentrations.

On the contrary, this occurs to the employees refueling cars during the refueling process, having as a consequence sometimes the quickly elevated levels of exposure to be hardly predicted with the same degree of accuracy.

The results of the third ANN are also very good, presenting similar evaluation values. This demonstrates that the model in its general form is very well fitted in filling stations with different characteristics of the emission sources. Having in mind the simplicity of the model (one input parameter fewer) and the fact that for both the filling stations one model is required, for applications aiming to predict the exposure levels of the employees, this is the intended model

### 3.6. Relative importance of the parameters constituting the exposure pattern obtained by the ANN model

**Figure 9.**
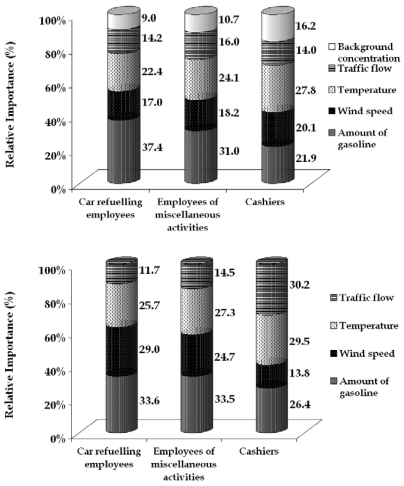
Relative importance of the parameters constituting the exposure pattern of the employees of urban (a) and rural (b) filling station respectively.

The main reason to apply two sets of ANNs (for each filling station) comes from the need to quantify the relative importance of the parameters that determine the exposure levels. Network modelling can assess the importance of each of the input variables by means of weights. The method for partitioning the connection weights proposed by Garson [31] was used. The technique involves partitioning the hidden–output connection weights of each neuron into components associated with each input neuron [32]. The results of the calculations are shown in Figures 9a-b, displayed as columns representing the relative importance of the various input variables. These results in combination with the active sampling ones clarify the relations of the emission sources (car refuelling and the corresponding amount of gasoline traded, direct traffic emissions and background concentration) with the meteorological parameters (wind speed and temperature).

In both filling stations, the amount of the fuel traded is the dominant parameter of exposure for employees dealing with car refuelling and miscellaneous activities. For cashiers the dominant exposure factor is either traffic flow or temperature depending on location.

The importance of traded fuel is decreasing proportionally to the distance from the fuel pumps. Thus this parameter is more important for employees refuelling cars, especially the ones working in the urban station, were the fuel amounts are higher.

Wind speed also affects the exposure of employees working outdoors. This is explained by the fact that the employees working outdoors are directly exposed to wind which influences the ambient benzene concentrations. The importance of the wind is stronger in the rural filling station because the dominant source emitting benzene (car refuelling) is not as strong as in the urban station and the ambient concentrations are more influenced by dispersion. Overall, for the employees working on refuelling wind speed is almost as important as the amount of gasoline traded; the difference between the two parameters is too small to be significant.

Temperature is a determining parameter, affecting fuel evaporation and consequently the presence of benzene in the air. The effect of temperature in both filling stations is stronger than the effect of wind, not only based on the daily variation, but also based in the average exposure values, that in summer are in general elevated. An exception to this pattern of relative importance occurs for the employees refuelling cars in the rural filling station.

Traffic flow in the road adjacent to the filling stations has a smaller effect than other parameters, because the dominant source of benzene in the filling stations has local origin. Some differences are observed in relation to distance from the road where the employees are located.

The urban station is larger; therefore, employees are farther away from the adjacent road than those from the rural one. On the contrary, the emission rate from the urban station adjacent road is significantly higher, resulting in increased importance compared to the rural one.

Finally, urban background concentration appears to have a small effect in the configuration of the exposure values, affecting mainly the cashiers. Background concentrations express the ambient benzene levels in the wider area, and these values respond slowly to the parameters affecting ambient benzene levels in a way similar to the exposure of cashiers. This parameter could be excluded from the network for simplicity without decisive loss in the accuracy of the predictions. However, if the model were applied in a filling station located in a more polluted area, background concentrations would need to be included in the ANN model.

### 3.7. Potential health risk estimation

The benzene concentrations measured in the environment and the related exposure levels do not constitute an acute health risk; thus, the main health concern is related to the possibility of leukemia incidences after chronic exposure such as the one characterizing occupational exposure to benzene. The estimated risk due to benzene exposure in the area under study is calculated considering:

- The exposure to benzene values obtained by the measurement campaign
- The related concentrations of benzene metabolites on target issue (in the case of leukaemia risk this is the bone marrow) through the developed PBPK/PD model and the related dose-response curve
- The variability of the population response through a Monte Carlo-Markov Chain approach

**Figure 10.**
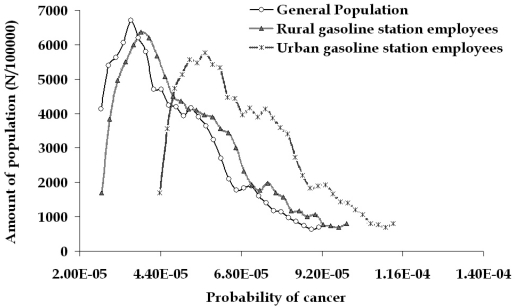
Estimated risk of leukaemia for the general population compared to the filling station employees (based on average exposure obtained by passive sampling)

The whole concept in developing a PBPK model is the use of a dynamic model for risk estimation, able to calculate possible interactions among the levels of benzene exposure through different activity patterns that may possibly lead to completely different concentrations of benzene and of the related metabolites in the blood and consequently to misjudged risk estimations. Furthermore, the model has the ability to calculate also the effects of toluene, xylenes and ethylbenzene to the metabolism of benzene. These compounds compete with benzene for CYP2E1, thus these co-exposures significantly reduce the overall metabolism of benzene relative to equivalent exposures of pure benzene. At low levels of exposure, competition for the P_450_ binding sites would be minimal and the same occur in the reduction of benzene's toxicity due to co-exposures. In the present study, both interactions mentioned above where extensively examined and under the exposure levels determined, no significant interactions where observed. The results of the calculations are presented in the probability distribution given in Figure 10. The obtained distributions represent the variation in activity patterns among individual employees and the variability of the population physiological response to environmental stressors.

Benzene toxicity is intimately tied to its complex metabolism and distribution. Key enzymes involved in the metabolism of benzene, including CYP2E1, the quinone reductase NQO1, and myeloperoxidase are polymorphic [33]. For example, a 7.6-fold difference in benzene-induced hematotoxicity in workers was observed among gene variants of CYP2E1 and NQO1. Thus the expression of genetic polymorphisms may modulate the sensitivity of an individual or ethnic group to the effects of benzene exposure.

The average estimated risk for the general population based considering exposure to benzene equal to 9 μg/m^3^ [30] is equal to 4.7·10^-5^, ranging from 2.4·10^-5^ to 9.4·10^-5^. For the filling station employees, risk calculation is slightly more complicated, because occupational exposure ***C_O_*** has to be inserted in the calculations. Assuming that people work for 30 years, for 5 days a week and 8 hours a day, and for the rest of their life span are exposed to the same concentrations ***C_C_*** of the control group, the average exposure ***C_A_*** for a 70 years life span is calculated through the equation:

$$C_{A}=C_{O}\cdot \frac{30y\cdot 5d\cdot 8h}{70y\cdot 7d\cdot 24h}+C_{C}\cdot \left(\frac{40y}{70y}+\frac{30y\cdot 2d}{70y\cdot 7d\cdot }+\frac{30y\cdot 5d\cdot 16h}{70y\cdot 7d\cdot 24h}\right)$$

Real-time estimation of personal exposure for all categories of employees allows us to take into account not only an average value of ***C_O_*** for the lifetime of a typical individual, but rather a cumulative ***C_O_*** that considers the variation in the effective biological dose of benzene metabolites due to the variation of personal exposure and ADME kinetics of benzene and its metabolites. Thus, over time a rather detailed account of personal exposure pattern due to occupational activities can be established. This pattern allows us to estimate the lifelong leukemia risk without having to resort to the introduction of hypotheses that may not consider properly short-term but repetitive variations of personal exposure, such as the ones associated with specific professional activities such as vehicle refueling. According to the above, the estimated risk for the occupational groups (as an average, minimum and maximum) for the urban gasoline station employees was estimated equal to 5.7·10^-5^, 3.1·10^-5^ and 11.2·10^-5^ (Figure 10).

**Figure 11.**
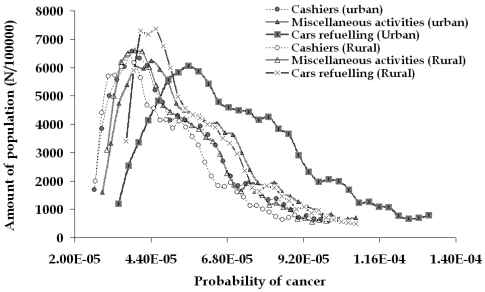
Estimated risk of leukaemia for the general population compared to the filling station employees based on the individual main activities (based on active sampling results).

For the rural gasoline station, the related risk is slightly lower and the corresponding values are equal to 5.2·10^-5^, 2.7·10^-5^ and 10.1·10^-5^. This distribution of leukemia probability is based on the average exposure levels of exposure for the employees of both filling stations. If the employees were continuously performing one kind of the three main activities, then three new distributions would be obtained (Figure 11), indicating the increased risk for the people dealing with car refueling (for example 7.0·10^-5^, 3.4·10^-5^ and 13.3·10^-5^ are the corresponding values of the urban filling station employees).

The results shown here demonstrate the usefulness of real-time estimation of personal exposure via the ANN algorithm implementation described above coupled with the PBPK/PD model that translates personal exposure values to health risk. The technology outlined in this paper can be readily used for chemical exposures where time dependence is important, such as for assessing acute neurotoxic effects or high exposure (e.g. emergency) situations. Nonetheless, it is useful even for assessing actual health risk of chronic effects such as induction of leukemia

## 4. Conclusions

Although the use of benzene is being progressively reduced over the last ten years, exposure to benzene still constitutes an environmental health risk due to the relatively elevated concentrations in several types of microenvironment, and in particular in occupational settings such as gasoline filling stations. The present study included field measurements as well as model development and evaluation for the purpose of building a computational platform, which uses environmental data that can be obtained by automated remote sensors and performs personal exposure assessment in real time for occupational groups such as filling station employees, coupled with calculation of the consequent health risks with particular reference to leukemia.

In order to perform these calculations, the determination of exposure is a critical step. The ANN developed based on the field measurements constitutes a reliable modeling technique with high quality results for this purpose as indicated by the evaluation parameters in Table 2. Not all parameters measured in the field measurement campaign (benzene concentrations) were measured by automated sensors. Yet, benzene concentration measurements are needed only to train the ANN model – they are not needed for the platform to run. All the other parameters required as input for the model (ambient temperature, wind speed, amount of fuel traded, traffic flow, urban background concentrations) were or can be easily determined by sensors. In terms of expandability and applicability of the model, the exposure model could be applied to any filling station as long as the field measurement campaign as described herein, in order to obtain the new weights for the input parameters that may differ significantly from area to area.

On the contrary, the PBPK model has no applicability limitations, as it does not need any recalibration, since its optimization is based on experimental data related to given values of exposure to benzene, while the link to pathology and the related estimated risk are based on in vitro toxicological studies [26, 28]. One other significant advantage of the PBPK model on that kind of applications is that it is a dynamic model, allowing fast and accurate response to changes of exposure concentrations, and consequently of the toxic metabolites at the target tissue (bone marrow) and finally to the estimated risk of chronic health effects such as leukemia, or acute effects such as neurotoxicity (the second most important adverse health outcome of benzene).

From the environmental policy perspective, the general concept of the platform proposed in this paper is of great interest, since it can be the basis of different applications in similar environmental settings, where personal exposure to contaminants is difficult to determine in real time but it is highly correlated to environmental parameters measured by automated real-time sensors. Being able to obtain in a cost-effective manner real-time exposure estimates facilitates the assessment of acute health effects such as neurotoxicity (evidentiated by symptoms such as vertigo, drowsiness, euphoria, headache, giddiness, narcosis, muscular incoordination, convulsions, paralysis, and unconsciousness) or respiratory conditions such as respiratory irritation, pulmonary edema, and pneumonia. Furthermore, meta-analysis of real-time risk estimation can indicate how to change the time-activity patterns of humans in specific microenvironments where human exposure is the highest in order to reduce the estimated risk down to acceptable levels. A typical example is given by the distinction among the distributions of estimated risk among the employees performing different activities in the same venue. For instance, on a day when both ambient temperature and the amount of fuel traded are elevated and calm wind conditions prevail, exposure to benzene and estimated risk are significantly increased for employees performing car refueling. In this case, shift interchange among employees (in the form of rotation) could be recommended as a real-time measure to reduce the cumulative health risk. Finally, taking into account the very short computation time needed (near real-time estimation), the integrated method outlined herein facilitates decision-making in the case of emergencies and optimization of risk management measures that are cost-effective, yet sufficiently protective of human health.

Future applications of this integrated computational platform will be geared towards developing the pathology models linking quantitatively benzene exposure to neurotoxic and respiratory effects in order to deliver a comprehensive tool that could be able to estimate both acute and chronic health risk of benzene using real-time personal exposure data. The proposed platform is a successful example of a scheme integrating different environmental sensors through data and model fusion algorithms in order to provide an effective tool for real time estimation of personal exposure and of the associated health risk to an occupationally exposed group. The algorithm structure requires a very small amount of environmental data (5 data classes used as input). This is due to the effectiveness of the individual models, which in turn is partially ascribed to the effectiveness of the Bayesian regularization algorithm in the development of models that describe linear and non-linear phenomena. Computational platforms like the one described in this study will be of increased interest in the forthcoming years due to the increasing public awareness about the interaction between environmental stressors and public health outcomes. We deem that health risk estimates given by similar approaches are more protective for occupational health than singly respecting ambient air limit values imposed by legislation and they can provide the basis for more cost-effective risk mitigation measures.

## Figures and Tables

**Table 1.** Percentage distribution of vehicle categories in each studied location.

	***Urban***	***Rural***
Catalytic	70	42
Non catalytic	8	11
Diesel vehicles	5	8
Light duty vehicles	7	23
Heavy duty vehicles	2	9
Buses	1	5
Motorcycles	7	2

**Table 2.** ANN exposure model evaluation parameters

		**Employees refueling cars**	**Miscellaneous Employees**	**Cashiers**
**RRMSE**	Urban	0.0678	0.0352	0.1081
	Rural	0.0880	0.0822	0.0501
	Urban + Rural	0.0900	0.0814	0.0556

**R^2^**	Urban	0.9729	0.9921	0.9379
	Rural	0.9437	0.9447	0.8136
	Urban + Rural	0.9714	0.9919	0.9441
